# Clinical outcomes of ankle fractures in sub-Saharan Africa: a systematic review

**DOI:** 10.1007/s00590-022-03397-7

**Published:** 2022-10-15

**Authors:** Peter Samuel Edward Davies, Rachel Pennington, Anil Singh Dhadwal, Linda Chokotho, Nohakhelha Nyamulani, Chiku Mpanga, Simon Matthew Graham

**Affiliations:** 1 Department of Trauma and Orthopaedics, Ninewells Teaching Hospital, Dundee, Scotland, UK; 2 The University of Dundee, Dundee, Scotland, UK; 3 The University of St Andrews, St Andrews, Scotland, UK; 4 Liverpool Orthopaedic and Trauma Service, Liverpool University Teaching Hospital Trust, Liverpool, UK; 5 Academy of Medical Sciences, Malawi University of Science and Technology, Thyolo, Malawi; 6 Trauma and Orthopaedics, Queen Elizabeth Central Hospital, BOX 95 Blantyre, Malawi; 7 Nuffield Department of Orthopaedics, Rheumatology & Musculoskeletal Sciences, Oxford Trauma and Emergency Care, University of Oxford, Oxford, UK; 8 Division of Orthopaedic Surgery, Groote Schuur Hospital, Cape Town, South Africa

**Keywords:** Ankle, Fracture, Africa, Outcome, PROM

## Abstract

**Purpose:**

Ankle fractures may cause disability and socioeconomic challenges, even when managed in a high-resource setting. The outcomes of ankle fractures in sub-Saharan Africa are not widely reported. We present a systematic review of the patient-reported outcomes and complications of patients treated for ankle fractures in sub-Saharan Africa.

**Methods:**

Medline, Embase, Google Scholar and the Cochrane Central Register of Controlled Trials were searched, utilising MeSH headings and Boolean search strategies. Ten papers were included. Data included patient demographics, surgical and non-surgical management, patient-reported outcome measures and evidence of complications.

**Results:**

A total of 555 patients with ankle fractures were included, 471 of whom were followed up (range 6 weeks–73 months). A heterogenous mix of low-quality observational studies and two methodologically poor-quality randomised trials demonstrated mixed outcomes. A preference for surgical management was found within the published studies with 87% of closed fractures being treated operatively. A total of five different outcome scoring systems were used. Most studies included in this review were published by well-resourced organisations and as such are not representative of the actual clinical practice taking place.

**Conclusion:**

The literature surrounding the clinical outcomes of ankle fractures in sub-Saharan Africa is sparse. There appears to be a preference for surgical fixation in the published literature and considering the limitations in surgical resources across sub-Saharan Africa this may not be representative of real-life care in the region.

## Introduction

It is estimated 90% of fractures worldwide occur in low- and middle-income countries (LMICs) [1]. Trauma healthcare services in LMICs are often ill-equipped to deal with the volume and complexity of injuries that present to them. If the outcomes of trauma care in LMICs were improved to be equivalent to high-income countries (HICs), it is estimated that over 2 million lives could be saved each year [2]. Disability arising from trauma causes a disproportionate burden on patients in LMICs where the results of inadequate trauma care are magnified by a lack of social welfare infrastructure and rehabilitation services [3]. Over 1 billion people live in sub-Saharan Africa (SSA), a region that contains predominantly LMICs [4]. The region bears a high burden of road traffic injuries with lower limb fractures occurring most commonly [5, 6]. Such injuries most frequently affect members of the working age population (18–44), leading to loss of employment for those who are commonly the primary source of income for a family and community [7].

Ankle fractures account for approximately 5% of all fractures presenting to healthcare in SSA [8, 9]. They are a heterogenous spectrum of injuries that require individualised approaches to management [10]. Some are stable and require only symptomatic management, whereas others are life-changing injuries which cause profound disability. Such disability may include chronic pain and altered gait biomechanics with subsequent difficulties in performing manual labour. The surgical management of ankle fractures can be challenging and requires significant skill with complication rates exceeding 36% even in well-funded institutions [11, 12]. Patients in SSA face inappropriate delays in accessing healthcare which is a particular problem with open ankle fractures and may result in poor outcomes [9]. Even when patients present timeously, hospitals may not have sufficient staffing or infrastructure to provide adequate care [13].

Defining the success of treatment is not always straightforward. Common complication rates such as infection and post-traumatic arthritis may be reported. Several functional patient-reported outcome measures (PROMs) have been developed in HICs [14]. The most commonly used outcome measure in foot and ankle surgery is the Olerud and Molander Ankle Score, followed by range of movement (ROM) and radiographic assessment [15]. No functional PROMs appropriate for use in ankle fractures have been specifically validated for use in LMIC populations.

To our knowledge, there are no systematic reviews describing ankle fracture management or outcomes in SSA. This review explores the literature surrounding outcomes of ankle fracture management in SSA, including surgical and non-surgical management, patient-reported outcome measures and evidence of complications and serves to summarise the current literature to aid direction for further research.

## Methods

This systematic review was written in accordance with the Preferred Reporting Items for Systematic Reviews and Meta-Analyses (PRISMA) statement [16]. The review protocol was registered prior to commencement with PROSPERO, the international register of systematic reviews database (CRD42021238654).

Inclusion criteria for the selection of studies were: (1) any study design; (2) full-text studies reported in English language; (3) any human patients of any age; (4) treated for ankle fracture; (5) treated in sub-Saharan Africa; and (6) reporting at least one type of outcome. Exclusion criteria were: (1) abstract-only publication; (2) Pilon-type ankle injuries; and (3) polytraumatised patients.

The primary outcome measures were any functional PROMs measured at follow-up and infection rates measured after 3 months minimum follow-up. Secondary outcome measures were: (1) non-union rates; (2) mal-union rates; (3) venous thromboembolism (VTE) rates; and (4) reoperation rates.

Three reviewers (PD, RP and AD) independently reviewed the literature following the same methodology. Medline, Embase, Google Scholar and the Cochrane Central Register of Controlled Trials were searched using all dates up to 14/03/2022. The above databases were accessed using Scopus, PubMed, Google Scholar and Cochrane websites. Citations were imported into Zotero reference management software. The full search strategy can be found as Appendix 1. Medical subject headings (MeSH) were combined using Boolean terms, synonyms, alternate spellings, and abbreviations. Relevant studies were backwards referenced. The final search was performed on 14/03/2022.

Information retrieved was: (1) country and publication year; (2) study population demographics including age and gender of patients, HIV positive status; (3) injury characteristics including closed or open injuries, classification of fracture types, classification of open injuries; (4) follow-up duration and rate; (5) function scores and PROMS; and (6) complications including infection, mal-union, non-union, VTE, reoperation rate.

Outcome data were extracted according to the follow-up defined in each study. The exact follow-up lengths including mean and range values were included when provided. Meta-analysis was planned only if populations, outcomes and follow-up were comparable. Simple baseline and outcome data were pooled when this was possible from the data extracted.

Risk of bias was assessed for each study by two authors (PD and RP). This was carried out using the Cochrane Collaboration guidelines using Risk Of Bias In Non-randomized Studies of Interventions (ROBINS-I) tool and the Risk of Bias tool (RoB 2.0) [17, 18]. The ROBINS-I tool scores observational studies that compare two or more interventions across seven domains (confounding, participant selection, classification of interventions, deviation from the intended intervention, missing data, measurement of outcomes, and selection of reported results) giving an overall judgement of “low”, “moderate”, “serious” or “critical” risk of bias. The RoB 2.0 tool scores randomised studies across five domains (randomisation, deviation from the intended intervention, missing outcome data, measurement of outcomes, selection of reported result) giving an overall judgement of “low”, “some concerns” or “high” risk of bias.

## Results

### Details of included studies

In total, 1150 papers were retrieved from our search. A PRISMA diagram demonstrating the search process is included in Appendix 2. Ten studies were included in the final analysis and are detailed in Table 1. Two studies were randomised controlled trials, three were prospective observational studies, and five were retrospective observational studies. Populations, outcomes and follow-up were not comparable, and therefore, no meta-analysis was completed.

**Table 1:** Studies of outcomes of ankle fracture in sub-Saharan Africa

Author [reference] (Date).Design *PMID*	Country *Economic classification by World Bank*	Enrolled Patients N *Classification*	Age.Mean (Range) [SD]	Female	HIV	Open Fracture N. *Gustilo Anderson classification*	Open fracture soft tissue coverage
Fonkoue L. [26]. (2021).Retrospective	Senegal.*Lower Middle Income*	52.*All fracture dislocations*	37.2.(19–68) [± 11.1]	22/52.42.3%	NR	NR	NA
Veldman F.J. [19]. (2020).Retrospective	South Africa *Upper Middle Income*	59.*All open fractures*	43.1.18–44 = 32.45–64 = 23. > 65 = 3.Unknown = 1	34/59.42%	Positive = 17.29%.Unknown = 3.5%.Negative = 39.66%	59.*I – 1.II – 43.IIIa – 7.IIIb – 5.IIIc – 0.Unknown – 3*	Primary closure = 11.Delayed primary closure = 4.Secondary intention = 10.SSG = 9.Flap = 0.Unknown = 25
Badenhorst D.H.S. [20]. (2020).Prospective.RCT	South Africa.*Upper Middle Income*	64.*All unstable*	42.9 [± 13.9].	32/51.63%	Positive = 2.4%.Unknown = 13.25%.Negative = 36.71%	NR	NA
Wichlas F. [13]. (2019).Retrospective	Sierra Leone.*Low Income*	14.*Not classified*	NR.*Demographics grouped with other injuries*	NR	NR	5.*NR*	NR
Muthuuri J.M. [25]. (2013).Prospective.RCT	Kenya.*Lower Middle Income*	62.*SER 39.PA 11.SA 7.PER 5*	41.(18–73).18–29 = 14.30–39 = 16.40–49 = 17.50–59 = 8.60–69 = 5. > 70 = 2	27/62.44%	NR	NR.*Grade I injuries included and combined with closed injuries for analysis*	NA
Kilonzo N. [27] (2014).Prospective	Kenya.*Lower Middle Income*	48.*Not classified*	47.(22–75)	29/48.60%	NR	3.*NR*	NR
Ogundele O.J. [21] 2013.Prospective	Nigeria.*Lower Middle Income*	70.*Weber A* = *9.Weber B* = *25.Weber C* = *36*	44.5.(17–80) [2.8] 10–19 = 2.20–29 = 8.30–39 = 11.40–49 = 29.50–59 = 14. > 60 = 6	30/70.43%	NR	21.*I* = *10.II* = *7.III* = *4.Grade IIIb and IIIc injuries excluded from study*	NR
Kuubiere C.B. [24] 2012.Retrospective	Ghana.*Lower Middle Income*	70.*Not classified*	36.(11–65) [9] 10–20 = 2.21–30 = 17.31–40 = 21.41–50 = 23.51–60 = 5.51–70 = 2	10/70.14%	NR	NR	NR
Bajwa A.S. [23] 2005.Prospective	South Africa.*Upper Middle Income*	52.*SER V* = *52*	48.(22–67)	29/52.56%	NR	*All open injuries excluded from study*	NA
Ngcelwane M.V. [22] 1990.Retrospective	South Africa.*Upper Middle Income*	64.*Weber A* = *7.Weber B* = *26.Weber C* = *24.Unknown* = *7*	NR.10–20 = 4.21–30 = 18.31–40 = 28.31–50 = 8. > 50 = 6	21/64.33%	NR	64.*NR.*Clean = 42.Contaminated = 22	NR

Treatment open fractures	Treatment closed or undefined Fractures	Open fracture infection rate	Closed fracture infection rate	Follow-up (Range) [SD]	Post–op outcome measure (Range) [SD]	Other complications
NA	ORIF 27.OREF 5.Mixed 2.Conservative18	NA	0/34	*N* = 44.27.2 months.(13–73) [± 18.3 months]	AOFAS.Mean 75.3 [± 15.8] (28–93). < 60 poor = 5.60–79 fair = 6.80–89 good = 25. > 90 excellent = 8	PTA OA 19
ORIF 21.Ex Fix 5.MUA + POP 1.K Wire 15.Combination 12.Unknown 5	NA	3/59.5%	NA	*N* = 21.@ 6 months	AOFAS.Mean 68.2.(38 – 95). < 60 poor = 9.60–79 fair = 6.80–89 good = 2. > 90 excellent = 4	NR
NA	ORIF 22.Fibula Nail 29	NA	1/51.2%	*N *= 51.@ 12 Months	OMFS.Median 100.(90–100).GRIMBY.Median 2.(2–2)	NR
NR	ORIF/Ex Fix 9.MUA + POP 2. POP 3.*All undefined*	1/5.20%	0/9	NR	NR	NR.*All complications except infection grouped with other injuries*
NR	ORIF 62.G1 Full WB.G2 Non WB	NR	Deep .1/65.2%.Superficial.2/65 .3%	*N* = 62.@ 6 Months	Modified.AAOS.Mean.G1 75.1 [15.6].G2 55.0 [12.0] P = 0.0001	Malunion 0.Loss of fixation 0
Ex Fix 3	ORIF 16.MUA + POP 29	1/3.33%	2/16.12.5%	*N* = 48.@ 6 Months	NR	Mal-union 10.Stiffness 12.Instability 8.Non-union 7.Osteoarthritis 5.Unstable fractures POP 92.3% complications Unstable fractures ORIF 37.5% complications
NR	ORIF 50.TBW 8.Ex Fix 4.Fusion 4.Combination 4	8/21.38%	2/49.4%	*N* = 70.Mean 18 Weeks.(6–20) [4.1]	OMFS.0–30 poor = 4.31–60 fair = 12.61–90 good = 12. > 91 excellent = 42	Wound dehiscence 2.Mal-union 6.Non-union 2
NA	ORIF 70	NR	0/70.0%	*N* = 70.@ 6 Months	NR	No complications
NA	ORIF 52.*All treated with lag screws and cerclage wire*	NA	0/52.0%	*N* = 52.@ 14 Weeks	NR	No complications
ORIF 27.K Wires 26.POP 11	NA	Deep .9/53.17%.Superficial.13/53.25%	NA	*N* = 53.Mean 12.3 Months (3–34)	Poor = 4.Fair = 7.Good = 19.Excellent = 23.	NR

NR = Not reported

NA = Not applicable

### Demographics of patients and injuries

A total of 555 patients with ankle fractures were included, 471 of which were followed up. Details of patient demographics are available in Table 1. Four papers were published from South Africa, two from Kenya, one from Sierra Leone, one from Senegal, one from Ghana and one from Nigeria. The economic classification as defined by the World Bank is included for each country. The median number of participants enrolled in each study was 60.5 (range 14–70). The mean age of patients included, across studies where this information could be clearly identified, was 42 years (range 11–75, *n* = 464). Two hundred and ninety-four patients were male (56%, range 37–86%, *n* = 528). Two papers reported the HIV status of patients (17% positive, 68% negative, 15% unknown, *n* = 110) [19, 20].

A classification of ankle injuries was available in three papers (*n* = 186). Two papers used the Danis–Weber classification, with 12% Weber A, 38% Weber B, 45% Weber C and 5% unknown (*n* = 134) [21, 22]. Two papers used the Lauge–Hansen classification with 80% supination external rotation type, 10% pronation adduction, 6% supination adduction and 4% pronation external rotation (*n* = 114) [23]. The Gustilo and Anderson classification of open fractures was used in two studies (*n* = 80), and this included 14% type I, 63% type II, 20% type III [19, 21]. Two papers did not report the inclusion or exclusion of open injuries [20, 24]. One paper included grade I open injuries but did not define the frequency of open injuries or analyse them as a subgroup [25]. The total number of open fractures included was 149 (range 2–64).

### Risk of bias

Assessment of bias was completed in three observational studies and two randomised studies [20, 24–27]. Assessment of bias was not possible using Cochrane Collaboration tools in one study as it did not compare two or more interventions and was simply a description of outcomes of a single intervention [23]. A breakdown of the seven domains is provided for each paper in Table 2 demonstrating that the included non-randomised studies were susceptible to significant bias and were of low quality. It was noted consistently that in the setting of SSA, patients are required to pay for their own treatments and often treatment type was decided by that which the patient could afford. There were generally insufficient analyses of confounding, and minimal mitigation strategies were reported. Table 3 demonstrates the five domains for the two randomised studies and shows the methodologies to give rise to “some concerns”. These were related to insufficient blinding of treatments among patients and investigators, and insufficient clarity surrounding the statistical plans and analyses.

**Table 2:** ROBINS-I assessment for bias in non-randomized studies of interventions

Author	Confounding	Participant Selection	Classification of Interventions	Deviation from intended Intervention	Attrition Bias	Detection Bias	Reporting Bias	Overall Risk of Bias
Fonkoue	Serious	Low	Moderate	No Information	Moderate	Moderate	Moderate	Serious
Veldman	Low	Low	Moderate	No Information	Moderate	Low	Moderate	Moderate
Wichlas	Serious	Low	Moderate	No Information	No Information	No Information	No Information	Grouped with other injuries
Kilonzo	Serious	Low	Moderate	No Information	Low	Moderate	Moderate	Serious
Ogundele	Serious	Low	Low	No Information	Low	Low	Moderate	Serious
Kuubiere*	Serious	Low	Moderate	No Information	Low	Moderate	Moderate	Serious
Bajwa								Not comparing treatments
Ngcelwane	Serious	Low	Moderate	No Information	No Information	Moderate	Moderate	Serious

*Kuubiere did not specify the mechanism by which patients were allocated to the normal ORIF group or the “modified” ORIF which they said was an advantage because it was cheaper

**Table 3:** RoB 2.0 assessment for bias in randomised trials

Author	Randomisation	Deviation from intended intervention	Missing outcome data	Measurement of outcomes	Selection of reported result	Overall Risk of Bias
Badenhorst	Low	Low	Low	Some concerns	Some concerns	Some concerns
Muthuuri	Low	Some concerns	Low	Some concerns	Some concerns	Some concerns

### Treatment

A summary of the treatment methods used in each study is provided in Table 1, along with details of open fracture management where this was identifiable. The treatment method was identifiable for 110 open fractures, with 44% of patients undergoing open reduction and internal fixation (ORIF), 37% Kirschner wires (K-wire), 7% External Fixation (Ex-fix), 1% manipulation under anaesthesia (MUA) and plaster of Paris (POP), 11% Combination. Only one paper reported on soft tissue management in open fractures with no use of any type of free or local skin flap surgery reported [19]. The management of 416 patients where the injury was closed or undefined consisted of 74% ORIF, 13% MUA and POP, 7% fibular nail, 2% tension band wiring, 3% Ex-fix, 1% fusion and 1% combinations of the above.

### Outcome scores

There was heterogenicity in the reporting of all outcomes between the papers. Six papers reported outcome scores, including AOFAS score, Olerud and Molander Functional Score (OMFS), Grimby scale, a modified AAOS score and an A-D score based on function [19–22, 25, 26]. No PROMs that were used in any of the papers were identified to have been validated within the population studied. All but one study defined the follow-up period at which PROMs were collected [13].

Veldman focussed on describing AOFAS scores when looking at the importance of anatomical reduction in the functional outcome of open ankle fractures, with a mean score of 68.2 at 6 months follow-up [19]. The authors noted that poor outcome scores correlated with failure of anatomical reduction of the fracture on factor analysis, resulting in statistical significance. In contrast, Badenhorst reported on OMFS and Grimby scale, with respective median scores of 100 and 2 at 12-month follow-up [20]. They found that no significant differences were found between the two treatment groups (ORIF vs fibula nail) at any time point.

Muthuuri reported a modified version of the AAOS score, which replaced “jogging” for “walking up stairs”, as they felt jogging was not an activity performed in their population [25]. A full explanation of the scoring system is detailed in the manuscript. Improved scores were seen in the full weight bearing group compared with non-weight bearing at 6 months (75 vs 55). Ogundele described OMFS and noted that 6% of patients had a poor outcome and each had suffered wound infection [21]. 77% of patients were found to have a good or excellent result. Kuubiere described 67.4% of patients achieving “full recovery” by 3 months and 100% achieving full recovery by 6 months [24]. No details were provided of the methods used to define “full recovery”. Ngcelwane reported on a classification according to function, which is described in the manuscript [22]. Group A is defined as “walking, pain free”, group B is “walking with pain on weight bearing”, group C is “requires a walking stick” and group D is “ankylosed joint or has arthrodesis”. Higher mean scores were described in the ORIF group compared to K-wires, but no statistical analysis was performed. 79% of patients were in groups A and B.

### Complications

Complication rates varied widely between papers. Details are provided in Table 1 along with breakdowns of complication types amongst closed and open fractures where these data were available.

#### Closed fractures

There were eight infections identified following operatively managed closed ankle fractures with an overall infection rate of 2.3% (range 0–12.5%, one deep, two superficial and five unspecified depth, *n* = 346). No infections were reported from conservatively managed closed ankle fractures.

Details of other complications were poorly reported overall. One paper reported 44% of patients as having post-traumatic ankle osteoarthritis at mean follow-up of 27.2 months but did not discriminate between operatively and non-operatively managed cases. No paper defined whether this was symptomatic arthritis or a radiographic-only diagnosis. Kilonzo et al. reported that 92.3% of unstable ankle fractures managed conservatively resulted in complications compared with 37.5% of operatively managed unstable fractures. Four papers did not define the presence or absence of complications other than infection at follow-up [19, 20, 22, 28].

#### Open fractures

There were 152 open fractures included in this review with 141 included with follow-up. There were 35 infections identified following open fractures (overall 25%, range 5–42%, *n* = 141). Ngcelwane did not report outcomes of 11 patients treated with POP for open ankle fractures, but specified between deep (25%) and superficial (17%) infection with an overall rate of infection of 42% in operatively managed open ankle fractures (*n* = 53) [22].

Veldman reported an infection rate of just 5% in 59 open ankle fractures managed operatively, but only 21 were followed up at 6 months [19]. No paper provided sufficient granularity of data to determine non-infectious complication rates in open fractures. No case of venous thromboembolism was reported in any case of open or closed fracture management.

## Discussion

There is a paucity of data describing the outcomes of ankle fracture management in SSA with the results of only 471 cases published. Only six of the ten papers included in this review are from PubMed listed journals; of these four are from South Africa which is an upper middle-income country, and the remaining two are from international authors working in non-governmental organisation (NGO) hospitals. The remaining papers were published in journals which are not PubMed indexed.

### Patient demographics

Patients undergoing management for ankle fractures in SSA were on average younger and more likely to be male, compared with data from HICs [29]. Patients in the UK were mean 50 years old compared with 42 years in SSA and were 60% female compared with 44% female in SSA. A systematic review of published trauma registry data shows that young male patients represent a large proportion of the LMIC trauma burden, especially patients involved in road traffic accidents [30]. These figures are likely related to the nature of their employment in the informal sector and inferior road safety initiatives in SSA.

### Treatment

In some HICs such as the UK, guidelines exist to demonstrate optimal care for patients with ankle fractures [29, 31]. Typically, more severe ankle fractures (which require realignment or are unstable) are treated with surgical management. Operative techniques described in the included papers were mostly in line with those practised in the UK with a preponderance for ORIF, external fixation and a fibula nailing system.

Only 11% of patients in the included papers were treated with casting which is lower than expected. A prospective registry study in Malawi previously identified that over 80% of patients were treated with casting [32]. Several papers specifically only included patients undergoing surgical fixation, and however, of the papers that included all treatments, the proportion undergoing cast treatment was low.

Data from LMICs suggest that when ankle fractures are treated operatively there is a reduction in the level of morbidity compared to those that are treated non-surgically [33]. Despite this, the majority of individuals experiencing ankle fractures across SSA, even if considered to require realignment, are thought to be treated non-surgically with a plaster cast which contradicts the published literature included in this review [34]. This is commonly performed by a non-physician with basic MSK training.

Standard plaster treatment often makes it difficult to achieve anatomical reduction, and even when realigned it is common for the fracture to re-displace. This can have major long-term consequences for the patient. Researchers in HICs are currently exploring the use of close contact casting in the management of unstable ankle fractures [35]. Researchers found that 81% of patients aged over 60 years could be managed in this way and had functional equivalence to patients undergoing primary surgical fixation, with cost savings. Research is currently in progress to apply CCC to patients age 18–60 years [36]. With adequate staff training, CCC could be a useful treatment method in SSA in the future as the material costs are minimal compared to performing surgical fixation.

### Outcomes

The outcomes described in the included papers were variable, with some examples of practice which may equate to that of HICs, and others far below the standards achieved in HICs, particularly relevant to infection rate in open ankle injuries with one paper describing a 38% infection rate [21]. Veldman reported on patients from the Ngwelezana Hospital, which is the main orthopaedic trauma referral centre for 15 district hospitals in the Northern KwaZulu-Natal province and performs 300 orthopaedic operations per month. Despite being in SSA this is a well-funded hospital in a UMIC which is likely to account for the good outcomes reported. Excluding this outlier, the infection rate from the other four papers is 35%.

Outcome scores were included in some papers, some showing near-perfect outcomes and other showing significant long-term functional impairment. There is a cost associated with retrieving PROMS data, which may explain why patient-reported outcomes were not widely used [37].

No PROMs were evidenced to have been validated previously in the SSA population which is an area for future development.

### Limitations

Ankle fractures are common injuries in SSA, and the true volume of surgical practice is not reflected in the volume of published data. The data included in this review represent a small sample, and the described studies are subject to significant methodological bias. There is also likely to be significant publication bias present, as organisations with higher levels of funding and better outcomes will have the resources to publish their results. Many of the papers in this review did not clearly report their data, and no studies reported use of the STROBE checklist [38].

Four papers included in this review were published using data from South Africa, which is a known outlier in terms of resources, in SSA. A further two papers were published by surgeons working in NGO hospitals with external funding. As such, the ten papers are not representative of a cross section of practice across SSA and represent work from some of the most well-funded institutions in the region. Studies included in this review were also limited to those in the English language. SSA has a high level of ethnic and linguistic variety, which may mean surgical teams across the region are ill equipped to publish in English-speaking journals [39].

We know from the previous literature that a large proportion of patients receive cast treatment for ankle fractures in SSA, but this was not demonstrated in the studies included in this review.

## Conclusions

The papers included in this review report data that is not likely to be truly representative of actual practice in SSA. Minimal data exist to describe the outcomes of conservative treatment for ankle fractures, which is likely be the method by which most of these injuries are treated. Therefore, we believe the current literature is not representative of the actual day-to-day clinical practice across SSA or demonstrates the true burden of ankle fractures across the region.

Future work should prioritise the accurate measurement of the burden of ankle fractures across SSA and determine how they are treated. The incidence of ankle fractures, mechanisms of injury and how they are treated should be known to allow appropriate resource allocation and planning of healthcare services for the management of these complex injuries. In low-resource settings, financial efficiency is important. Educating staff in the use of simple interventions, such as CCC, could enable them to provide higher quality care for a large proportion of patients with ankle fractures, at a reduced cost compared with investment in advanced surgical facilities. This is a potential area of future research.

## Appendix 1: Search strategy for use in database searches

(Fracture) AND ((malleolus OR malleolar OR ankle) AND (angola OR ethiopia OR niger OR benin OR gabon OR nigeria OR botswana OR gambia OR rwanda OR (burkina AND faso) OR ghana OR (são AND tomé AND principe) OR burundi OR guinea OR senegal OR (cabo AND verde) OR (guinea-bissau) OR seychelles OR cameroon OR kenya OR (sierra AND leone) OR ( central AND african AND republic) OR lesotho OR somalia OR chad OR liberia OR africa OR comoros OR madagascar OR ( south AND sudan) OR congo OR malawi OR sudan OR congo OR mali OR tanzania OR ( côte AND d'ivoire) OR mauritania OR togo OR ( equatorial AND guinea) OR mauritius OR uganda OR eritrea OR mozambique OR zambia OR eswatini OR namibia OR zimbabwe).

## Appendix 2: PRISMA 2020 flow diagram for systematic reviews

Page MJ, McKenzie JE, Bossuyt PM, Boutron I, Hoffmann TC, Mulrow CD, et al. The PRISMA 2020 statement: an updated guideline for reporting systematic reviews. BMJ 2021;372:n71. https://doi.org/10.1136/bmj.n71. For more information, visit: http://www.prisma-statement.org/.
