# Correction to: Clinical outcomes of ankle fractures in sub-Saharan Africa: a systematic review

**DOI:** 10.1007/s00590-022-03423-8

**Published:** 2022-11-28

**Authors:** Peter Samuel Edward Davies, Rachel Pennington, Anil Singh Dhadwal, Linda Chokotho, Nohakhelha Nyamulani, Chikumbutso C. Mpanga, Simon Matthew Graham

**Affiliations:** 1 Department of Trauma and Orthopaedics, Ninewells Teaching Hospital, Dundee, Scotland, UK; 2 The University of Dundee, Dundee, Scotland, UK; 3 The University of St Andrews, St Andrews, Scotland, UK; 4 Liverpool Orthopaedic and Trauma Service, Liverpool University Teaching Hospital Trust, Liverpool, UK; 5 Academy of Medical Sciences, Malawi University of Science and Technology, Thyolo, Malawi; 6 Trauma and Orthopaedics, Queen Elizabeth Central Hospital, BOX 95, Blantyre, Malawi; 7 Queen Elizabeth Central Hospital, Blantyre, Malawi; 8 Nuffield Department of Orthopaedics, Rheumatology & Musculoskeletal Sciences, Oxford Trauma and Emergency Care, University of Oxford, Oxford, UK; 9 Division of Orthopaedic Surgery, Groote Schuur Hospital, Cape Town, South Africa

**Correction to: European Journal of Orthopaedic Surgery & Traumatology** 10.1007/s00590-022-03397-7

The original version of this article unfortunately contained a mistake. One of the author’s name and affiliation are incorrect. The correct author’s name and affiliation should be

Chikumbutso C. Mpanga

Queen Elizabeth Central Hospital, Malawi.

